# Quantitative Susceptibility Mapping in Skull Base Chordoma: In Silico Analysis and In Vivo Application Towards Indirect Hypoxia Assessment

**DOI:** 10.1002/mrm.70193

**Published:** 2025-11-24

**Authors:** P. Fenech, L. Morelli, G. Parrella, S. Imparato, A. Iannalfi, S. Lillo, E. Orlandi, G. Baroni, C. Paganelli

**Affiliations:** ^1^ Dipartimento di Elettronica, Informazione e Bioingegneria Politecnico di Milano Milano Italy; ^2^ Centro Nazionale di Adroterapia Oncologica Pavia Italy; ^3^ Department of Internal Medicine and Therapeutics University of Pavia Pavia Italy; ^4^ Department of Clinical, Surgical, Diagnostic, and Pediatric Sciences University of Pavia Pavia Italy

**Keywords:** hypoxia, in silico validation, particle therapy, quantitative MRI, quantitative susceptibility mapping, skull base chordoma

## Abstract

**Purpose:**

To evaluate quantitative susceptibility mapping (QSM) beyond the brain through realistic simulations and to explore preliminary evidence that may be indicative of hypoxia in skull base chordomas (SBC).

**Methods:**

Each step of the QSM pipeline was optimized within an in silico framework consisting of (i) phase unwrapping, (ii) background field removal, and (iii) dipole field inversion, which were tested on a realistic phantom to generate accurate susceptibility maps. The optimized pipeline was then applied to seven SBC patients, analyzing tumor heterogeneity and correlating QSM features with the proliferation index (Ki‐67), towards hypoxia assessment. A binary classifier was developed to distinguish low‐ and high‐proliferation tumors based on first‐order QSM features.

**Results:**

The optimal phase unwrapping method combined with dipole inversion provided an error of 38.36 ppm. The best strategy for background field removal exhibited the lowest error (from 49 to 53 Hz). In SBC patients, tumor heterogeneity was observed, and a statistically significant correlation (*p* < 0.05) was measured between Ki‐67 versus QSM maximum value and interquartile coefficient of variation within the tumor volume (Spearman's coefficients of 0.8 and −0.8, respectively). The classifier achieved 85.7% accuracy.

**Conclusion:**

This study provides a foundation for characterizing SBC through QSM, enabling indirect, non‐invasive identification of potentially hypoxic tumor regions. Further histological validation with specific hypoxia markers, such as HIF‐1α, is nevertheless required.

## 1 Introduction

Hypoxia, an oxygen‐deficient condition that drives tumor progression and confers radioresistance, is a critical factor to assess for the optimization of radiotherapy [1]. Particle therapy with protons or carbon ions is particularly effective against hypoxic, radiation‐resistant tumors [2], owing to its high linear energy transfer and precise dose deposition via the Bragg peak [3]. However, its potential is limited by the lack of reliable, non‐invasive methods for detecting hypoxia and evaluating its prognostic value.

Skull base chordoma (SBC), a rare and aggressive bone sarcoma, is resistant to chemotherapy and conventional radiotherapy [3], with surgery often limited by its proximity to critical structures [4]. Consequently, particle therapy has become the preferred post‐surgical treatment option [5]. Preliminary evidence suggests that surgical residuals of SBC monitored by ^18^F‐fluoromisonidazole (^18^FMISO)‐PET‐CT may contain hypoxic regions, which potentially contribute to an increased radio‐resistance [6].

Unlike ^18^FMISO PET [7] or oxygen‐enhanced MRI (OE‐MRI) [8], which require complex acquisition protocols or contrast agents, gradient‐echo (GRE) multi‐echo MRI could offer a simpler, non‐invasive alternative by exploiting magnetic susceptibility. GRE sequences are highly sensitive to susceptibility and underpin techniques such as Quantitative Susceptibility Mapping (QSM) [9]. Whereas T2* imaging [10] and Susceptibility Weighted Imaging (SWI) [11] reflect combined effects from adjacent tissues, QSM isolates local susceptibility for precise, region‐specific measurements.

Key susceptibility biomarkers include iron, in its heme and non‐heme forms, and calcium [9, 12]. Non‐heme iron supports neuronal functions [13], while heme iron forms hemoglobin for oxygen transport. Hemoglobin's susceptibility varies with oxygenation: *oxyhemoglobin* is diamagnetic, whereas *deoxyhemoglobin* is paramagnetic, a hallmark of hypoxia. Given these susceptibility changes, QSM has been applied as an indirect marker of hypoxia on healthy subjects [14].

The QSM processing involves (1) the combination of the raw phase images with phase‐offsets correction, (2) phase unwrapping, (3) the background field removal, and (4) the dipole field inversion. Over the years, significant improvements have been achieved for phase‐offset correction [15] and now it can be handled with an appropriate coil combination method during the acquisition. Phase unwrapping instead is critical for recovering the correct phase values from the measured phase, which is constrained to a range between −*π* and *π*. The outcome of this operation is a total field map that includes background contributions from magnetic sources located outside the region of interest. The background field removal aims to separate these background fields from the local field generated within the ROI. Lastly, dipole field inversion is performed to calculate the distribution of magnetic susceptibility in tissues, based on the local magnetic field map, thus providing the final local susceptibility map.

Originally developed for neuroimaging, QSM has recently expanded to other organs [16], including prostate [17], liver [18], breast [19], kidney [20], cardiovascular [21] and musculoskeletal systems [22]. While showcasing its versatility, these applications pose technical challenges due to organ‐specific anatomy. Nonetheless, most QSM studies still focus on the brain, where optimized pipelines help minimize artifacts and noise. Accordingly, in conventional neuroimaging protocols, the region of interest (ROI) is typically restricted to intracranial brain structures, with additional erosion of the brain mask performed [23] to avoid areas prone to artifacts, such as tissue‐air and tissue‐bone interfaces. Extending QSM to regions like SBC is challenging due to sharp susceptibility contrasts at bone–tissue interfaces, which complicate accurate mapping.

In light of these challenges, the aim of the present study is to develop an effective pipeline for generating QSM maps that extend beyond the brain, adapting existing algorithms for the specific application to the skull base region. Specifically, the performance of various algorithms for QSM post‐processing step is evaluated through in silico simulations, involving a realistic simulation of the entire head of a patient. Finally, susceptibility maps of a preliminary cohort of seven patients with SBC are analyzed before particle therapy. Correlation with the cellular proliferation index (Ki‐67 [24]) is performed under the hypothesis that QSM‐derived signal may reflect hypoxic tumor regions [25, 26], and simple predictive models are tested using selected QSM‐derived features to evaluate the potential of QSM for indirect, non‐invasive hypoxia estimation in SBC.

## 2 Theory

### 2.1 Phase Unwrapping

The signal of the magnetization vector in the complex plane comprises two components: the magnitude and the phase, that represent the length and the angle of the vector, respectively. Physically the phase changes linearly in time depending on the field variation induced by the magnetic local susceptibility, following the equation

(1) $$\varphi =\gamma ∆B\;T_{E}$$

where γ is the gyromagnetic ratio, ∆B is the magnetic field variation induced by susceptibility and $T_{E}$ is the echo time. However, the phase is measured only in a range between −π and π, leading to the wrapping phenomenon. Therefore, to compute the induced magnetic field, it is necessary to reconstruct the phase by phase unwrapping.

Unwrapping methods can be broadly categorized into approximate and exact methods. Laplacian methods are approximate and can cause errors near phase discontinuities, while best path and region‐based methods are exact, resolving 2*π* ambiguities to recover the true phase. Exact methods like ROMEO [25] and SEGUE [26] (a successor to PRELUDE [27]) yield fewer unwrapping errors, particularly in challenging areas such as veins and hemorrhages, thereby improving QSM accuracy in oxygenation estimation [28]. Given the importance of precise susceptibility mapping for this application, we focused our study on these exact methods (Supporting Information S1).

### 2.2 Background Field Removal

The distinction between internal and external fields is based on the physical location of the susceptibility sources. In a first‐order approximation, the internal and external background fields are considered independent of each other and are generated by susceptibility sources inside and outside the ROI, respectively [29]. The total magnetic field estimated by the unwrapped phase is defined as:

(2) $$B_{\mathrm{tot}}=B_{\mathrm{loc}}+B_{\text{back}}$$

where $B_{\mathrm{loc}}$ and $B_{\text{back}}$ are magnetic fields induced by susceptibility sources located inside and outside the ROI, respectively. Several methods have been proposed to remove the background field effects and accurately estimate the local susceptibility map: in studies focused solely on the brain, erosion at the edges is commonly implemented directly on the brain to prevent distortion and facilitate background field removal. However, at interfaces between materials with different susceptibility (e.g., air‐tissues and skull‐tissue), additional boundary fields are generated that cannot be solely attributed to internal or external sources [23].

In this study, we compared RESHARP [30], VSHARP [31], PDF [32], and LBV [33] methods (Supporting Information S2), evaluating each method's ability to handle susceptibility variations at the skull interface through simulations modeling relevant anatomical and physical effects.

### 2.3 Dipole Field Inversion

The relationship between the magnetic field distortion and the susceptibility is given by

(3) $$\delta B=F^{-1}DF\chi$$

where F and $F^{-1}$ denote the forward and inverse Fourier transform, D is the unit dipole kernel in Fourier space and χ represents the susceptibility. The dipole inversion problem, which involves deconvolving the measured local field with the unit dipole kernel, is ill‐posed due to zero and small values in D, which leads to non‐uniqueness and instability in the solutions. To address this problem and ensure a stable solution, some approaches introduce strategies of regularization. Among them, we selected the Least‐norm QSM (LN‐QSM) with Tikhonov regularization method [34]. This method is optimized for extracting the susceptibility of the whole head, integrating the Tikhonov regularization with a Total Variation regularization (TV) to denoise the image. Specifically, for the purpose of this study, we applied the automated whole‐head reconstruction approach, following the thresholding‐based algorithm, which automatically generates a binary soft‐tissue mask and computes the susceptibility map for brain and non‐brain regions. This approach solves the following minimization problem

(4) $${\text{argmin}}_{\chi }{\left\|W(M_{s}\;F^{-1}D\;F\chi -M_{s}\delta B\right\|}_{2}^{2}+\lambda_{1}\;\mathrm{TV}\left(\chi \right)+\lambda_{2}\;{\left\|WM_{s}\;\chi \right\|}_{2}^{2}$$

where $M_{s}$ is the soft tissue mask, TV term is $\lambda_{1}$, Tikhonov regularization term is $\lambda_{2}$ and W is a data weighting factor extracted from the first echo's magnitude image divided by the mean magnitude intensity of the brain region.

## 3 Methods

**FIGURE 1:**
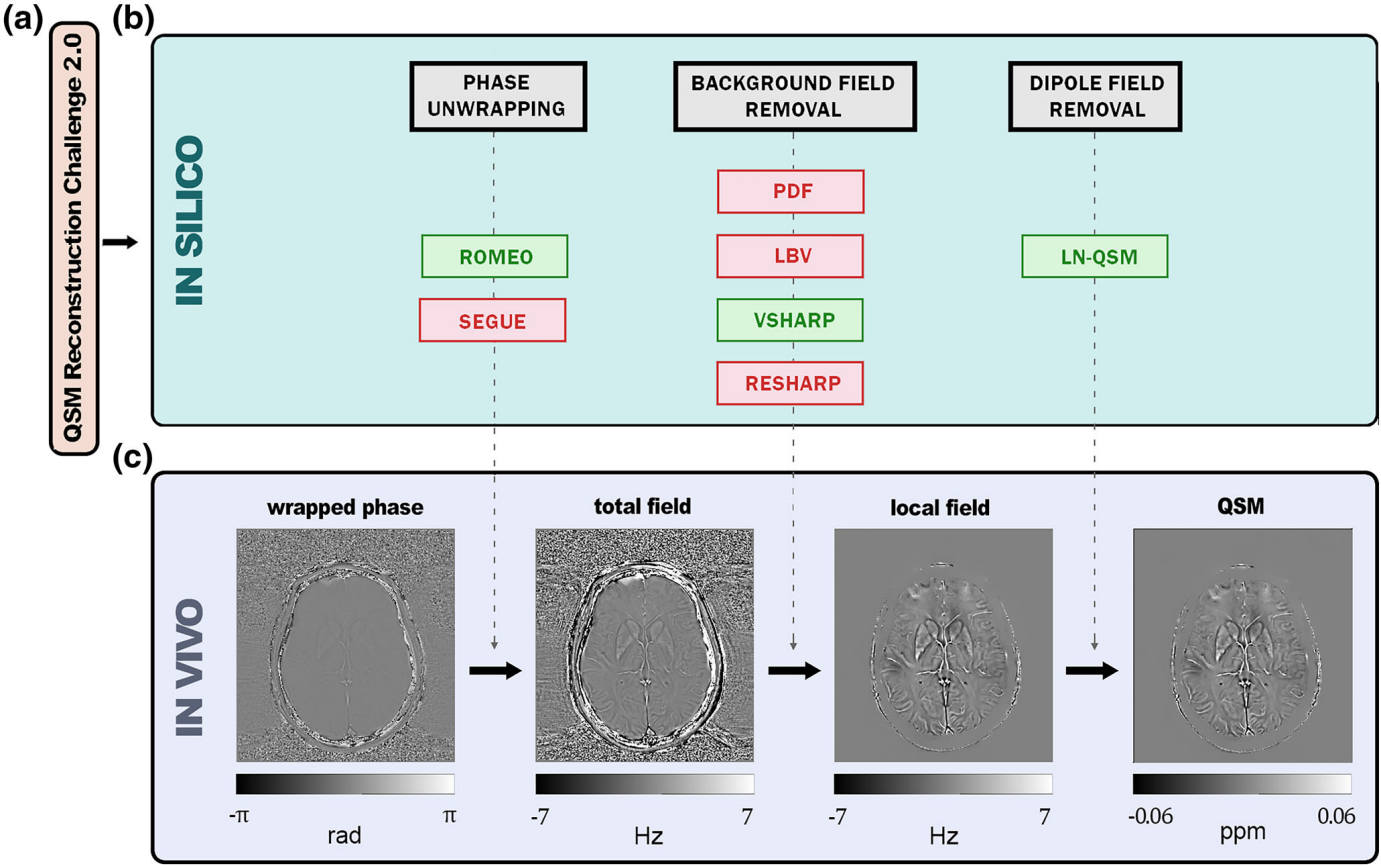
Complete pipeline illustrating the QSM processing workflow, organized into three sequential sections: (a) the QSM Reconstruction Challenge 2.0 dataset, used as input for the simulation framework; (b) the in silico evaluation, where tailored simulations derived from A allowed testing of multiple methods at each step of the QSM pipeline—selected algorithms are highlighted in green, while others are in red; and (c) the in vivo application, where the optimized pipeline was applied to real MRI patients' data.

Figure 1 reports the workflow of the methodology for in silico (Figure 1a,b) and in vivo analyses (Figure 1c). All data processing was performed in MATLAB (version 2023b, The MathWorks, Natick, MA, USA) and Python (version 3.11.5, Python Software Foundation, Wilmington, DE, USA) on a laptop (Intel Core i7‐12650H) with 16 GB RAM.

### 3.1 In Silico Simulation

#### 3.1.1 In Silico Simulation Framework

**FIGURE 2:**
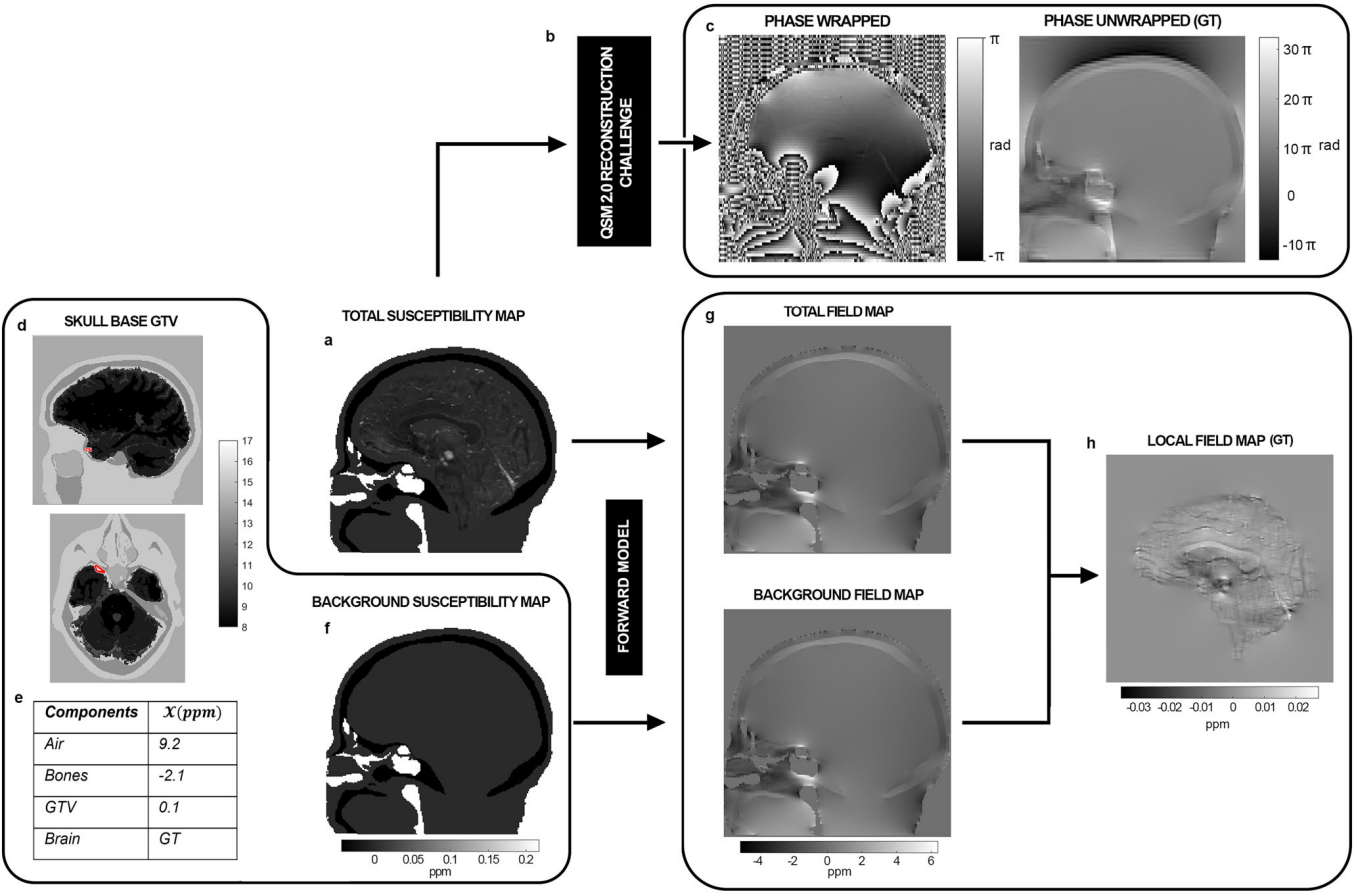
The three simulations developed to represent the main steps of the QSM pipeline are organized with inputs on the left, signal simulation models in the center, and output data on the right. Throughout the figure, the label “GT” denotes ground truth. The total susceptibility distribution (a), shared across all simulations, is provided to the signal simulator used in the QSM Reconstruction Challenge 2.0 (b) to generate magnitude and phase images. Panel (c) represents the first simulation that includes background field contributions. The third simulation, following the same workflow but excluding background fields, corresponds to the challenge dataset provided to participants and is shown in Figure S1. In the second simulation, focused on background field removal, the susceptibility map was enriched with a synthetic GTV, segmented on anatomical structures and outlined in red (d). The assigned susceptibility values are listed in the table (e), where “GT” refers to the magnetic susceptibility values of the brain labeled as ground truth in the original challenge dataset. The forward model applied to both the full and background‐only susceptibility distributions (f) allows simulation of the total and background fields (g), from which the local field is finally obtained (h).

The open source QSM Reconstruction Challenge 2.0 dataset [35, 36] served as the control case for our simulation framework (Figure 1a). This dataset provided a realistic, non‐homogeneous acquisition comprising magnitude and phase data of the entire head in absence of background fields, detailed anatomical segmentation, and ground truth magnetic susceptibility maps (Supporting Information S3 and Figure S1). We customized the dataset for three simulation settings to test key components of the QSM pipeline (Figures 1b and 2). The specific simulation procedures adopted for each case are described in detail in the following sections.

#### 3.1.2 Phase Unwrapping

In the first simulation, phase unwrapping was tested using realistic conditions. The total magnetic susceptibility map (Figure 2a) was provided as input to the open‐source signal simulator developed for the QSM Reconstruction Challenge 2.0 (Figure 2b), which computes complex MR signals from a given susceptibility distribution while explicitly accounting for acquisition parameters. Since the acquired phase is not a simple linear scaling of the total field, this simulator was selected to enable realistic reconstruction of both magnitude and phase under conditions reflecting our dataset. In this implementation, we enabled the inclusion of background field contributions, absent in the official challenge dataset, to simulate a wrapped phase (Figure 2c), along with the corresponding magnitude image. This wrapped phase served for testing the unwrapping algorithms ROMEO and SEGUE, whose estimation was compared with respect to the ground truth unwrapped phase, extracted by the simulation process.

To ensure comparability and consistency with our in vivo dataset, the simulation was homogenized using the same acquisition parameters: a main magnetic field aligned with the *z*‐axis, a field strength of 3 T, resolution of 0.75 × 0.75 × 2.5 mm^3^, flip angle (FA) of 20°, repetition time ($T_{R}$) of 61 ms, echo times ($T_{E}$) of 4, 8, 12, and 16 ms. A null phase offset was assumed.

Root Mean Square Error (RMSE) and Mean Absolute Error (MAE) were adopted to quantify the performance of the unwrapped methods. Specifically, the evaluation focused on local spatial phase variation within two ROIs: the entire brain volume and a peri‐bone shell were defined by expanding the bone interface through 3D morphological dilation using a spherical kernel (diameter: 13 voxels). This peri‐bone region, where phase unwrapping is particularly challenging due to strong susceptibility variations near bone structures, corresponds to an approximate spatial thickness of 4.5 mm in the *x*–*y* plane and 15 mm along the *z*‐axis, consistent with the voxel resolution.

Additional analyses were subsequently performed to assess noise robustness and computational performance of the selected methods on complex, extended geometries (Supporting Information S4).

#### 3.1.3 Background Field Removal

The second simulation was designed to evaluate background field removal methods in a setting that includes all relevant field‐contributing components, thereby mimicking clinical anatomical complexity. For this purpose, we modified the original total susceptibility map by adding a synthetic Gross Tumor Volume (GTV) with a fictitious susceptibility value (Figure 2d). The GTV was manually placed in a representative skull base location, reflecting the anatomical and spatial features of our clinical cohort. Susceptibility values assigned to each structure are summarized in Figure 2e.

In this simulation we implemented the Forward Model [37], a framework used to simulate the magnetic fields induced by a given susceptibility distribution. This model was applied to both the total and background‐only susceptibility maps (Figure 2f) to simulate total and background magnetic fields (Figure 2g). The local field, representing the ground truth to test background field removal models, was finally computed by subtraction, following the reference scan method [32] (Figure 2h). The Forward Model simulation assumed a main magnetic field aligned with the *z*‐axis, a field strength of 7 T, isotropic resolution of 1 × 1 × 1 mm^3^, matrix size of 164 × 205 × 205 voxels.

RMSE and MAE were computed between the ground truth local field map and the one estimated by the background field removal methods—VSHARP, RESHARP, LBV, and PDF. Additionally, the robustness of the background field removal methods as a function of the degree of erosion was evaluated as follows. Starting from a ROI including the whole head and excluding bones and air, the extent of erosion was progressively increased by modifying a specific parameter in each algorithm: (1) in RESHARP, the radius of the spherical kernel used for computing the Spherical Mean Value (SMV) was progressively increased, starting from a single voxel (i.e., minimal erosion); (2) in VSHARP, the minimum kernel radius was incrementally increased from one voxel, while maintaining a fixed maximum radius of 10 voxels; (3) in LBV, the number of voxels involved in the peel operation was increased from an initial value of 1, while the depth parameter remained fixed at 3; (4) in PDF, the number of eroded voxels was adjusted prior to the application of the function. All other parameters of each algorithm were kept constant throughout the evaluation.

Varying the different parameters in each method allows for different degrees of erosion. We quantified this by computing the percentage of eroded voxels over the ROI mask encompassing the brain and GTV, as well as on the GTV alone. It should be noted that a direct comparison of erosion factors is not feasible, as this depends on the varying parameters specific for each algorithm. Since most of the selected methods involve minimal erosion that removes part of the GTV located at the ROI boundary, we also evaluated the impact of an a priori expansion to compensate for this effect.

An additional 3D polynomial fitting, as well as spherical harmonics up to the fourth order, were tested to further mitigate residual background field contributions that persisted after standard background removal techniques (Supporting Information S5).

#### 3.1.4 Dipole Field Inversion

The third simulation refers to the official dataset provided by the QSM Reconstruction Challenge 2.0, which was generated by the organizers and made available to participants for benchmarking dipole inversion algorithms. This dataset consists of a total magnetic susceptibility distribution as input, with the complex MR signal computed under idealized conditions excluding background field effects. We evaluated the performance of the dipole inversion using the provided brain susceptibility maps as ground truth (Figure S1).

Dipole inversion was performed using the LN‐QSM method, with regularization parameters set according to the original publication: TV term to $\lambda_{1}=4\times 10^{-4}$, the Tikhonov regularization term to $\lambda_{2}=1\times 10^{-3}$, $M_{s}$ as the soft tissue mask and W derived from the first echo's magnitude image divided by the mean magnitude intensity of the brain region.

In addition to RMSE, the Normalized Root Mean Square Error (NRMSE) and MAE were also computed separately for different anatomical structures (Figure S1c). Specifically, the following structures were considered: caudate, globus pallidus (GP), putamen, red nucleus (RN), dentate nucleus (DN), substantia nigra & sub‐thalamic nucleus (SN and STN), thalamus, white matter (WM), gray matter (GM), cerebro spinal fluid (CSF), blood, fat and muscle.

### 3.2 In Vivo SBC Assessment

#### 3.2.1 SBC Data

Seven patients with SBC were scanned using a 3 T scanner (Skyra, Siemens Healthineers, Erlangen, Germany) at the National Centre for Oncological Hadrontherapy (CNAO, Pavia, Italy). The study received approval by the local Ethical Committee and informed consent was obtained from patients.

Data were acquired at baseline (i.e., before particle therapy treatment) with a 3D multiple Gradient‐Echo (GRE) via the Adaptive Combine coil combination method on a 32‐channel head coil. The acquisition parameters were as follows: a FA of 20°, a $T_{R}$ of 61 ms, the first and last $T_{E}$ were set 4.35 and 15.39 ms with an echo spacing of 1.84 ms, the bandwidth was 680 Hz/voxel and the spatial resolution was 0.75 × 0.75 × 2.5 mm^3^. GRAPPA with an acceleration factor of 2 was applied. The average total scan time was 5 min and 40 s.

The GTV structures, contoured on CT scans as per clinical practice, were co‐registered on the GRE following a multi‐resolution registration pipeline, using Plastimatch v.1.9.0 [38].

Diffusion‐weighted imaging (DWI) scans were also acquired with an echo planar diffusion‐weighted pulse sequence applied along three orthogonal directions, with 12 optimized b‐values [39]. Key acquisition parameters included: a FA of 90°, a $T_{R}$ of 300 ms, an $T_{E}$ of 65 ms with a spatial resolution of 1.15 × 1.15 × 4.0 mm^3^, and a slice gap of 0.8 mm. The *b* = 0 s/mm^2^ image was acquired in both anterior‐to‐posterior and posterior‐to‐anterior directions to correct for distortion. DWI distortion was corrected with FSL TOPUP [40] on *b* = 0 s/mm^2^ volumes, then registered to the GRE volume. Multiplicative Intrinsic Component Optimization (MICO) algorithm [41] was used to segment White Matter (WM), Gray Matter (GM), and Cerebrospinal Fluid (CSF).

#### 3.2.2 In Vivo Quantitative Analyses

QSM maps were derived from GRE acquisitions. After phase unwrapping, regions with high susceptibility variations, such as ears or cranial air cavities, were excluded and a dilation of one voxel to the GTV mask was performed. Susceptibility maps were then reconstructed using dipole field inversion, completing the pipeline (Figure 1c).

Quantitative evaluations involved comparing susceptibility values between GTV and healthy structures, including WM (considered as reference), GM and CSF (Kruskal–Wallis test, alpha = 5%), as well as investigating the relationship between susceptibility values and the Ki‐67 proliferation index (Spearman correlation, alpha = 5%). Analyses were conducted on both first order and volumetric features of the QSM map (Supporting Information S6 and Table S1). The correlation analysis was conducted across all GTV voxels, as well as separately for paramagnetic and diamagnetic subsets, defined according to the physical sign of the susceptibility values: voxels with positive (χ > 0) and negative (χ < 0) values, respectively.

To classify tumors by Ki‐67, we developed a binary classifier using first‐order QSM features, based on the threshold by Morelli et al. [42] (Ki‐67 < 5%: low, ≥ 5%: high), with four high‐ and three low‐proliferation cases. To minimize overfitting due to the small sample size, two modeling scenarios were tested (additional details in Supporting Information S6): a minimal model with two features that were significantly correlated with Ki‐67 (*p* < 0.05, Spearman ≥ 0.8) and not redundant (inter‐feature correlation < 0.7), and an extended model with a third feature automatically selected via Recursive Feature Elimination (RFE). Feature scaling was applied with zero mean and unit variance.

Model training followed a nested cross‐validation setup: a three‐fold inner loop for hyperparameter tuning (Grid Search) and an outer Leave‐One‐Out (LOO) loop for performance evaluation on unseen data. Classifiers tested included a simple linear model (logistic regression), a kernel‐based method (Support Vector Machine [SVM]) and two tree‐based ensemble methods (random forest and gradient boosting) as they represent four complementary approaches.

Performance was assessed using accuracy, precision, recall, and *F*1‐score [43]. Feature importance was also evaluated for each model. In random forest, it was based on the average decrease in Gini impurity, reflecting each feature's contribution to node purity, with higher values indicating greater predictive relevance. For logistic regression and SVM classification, importance was derived from the absolute values of the model coefficients, normalized to yield relative scores between 0 and 1.

## 4 Results

### 4.1 Phase Unwrapping

**FIGURE 3:**
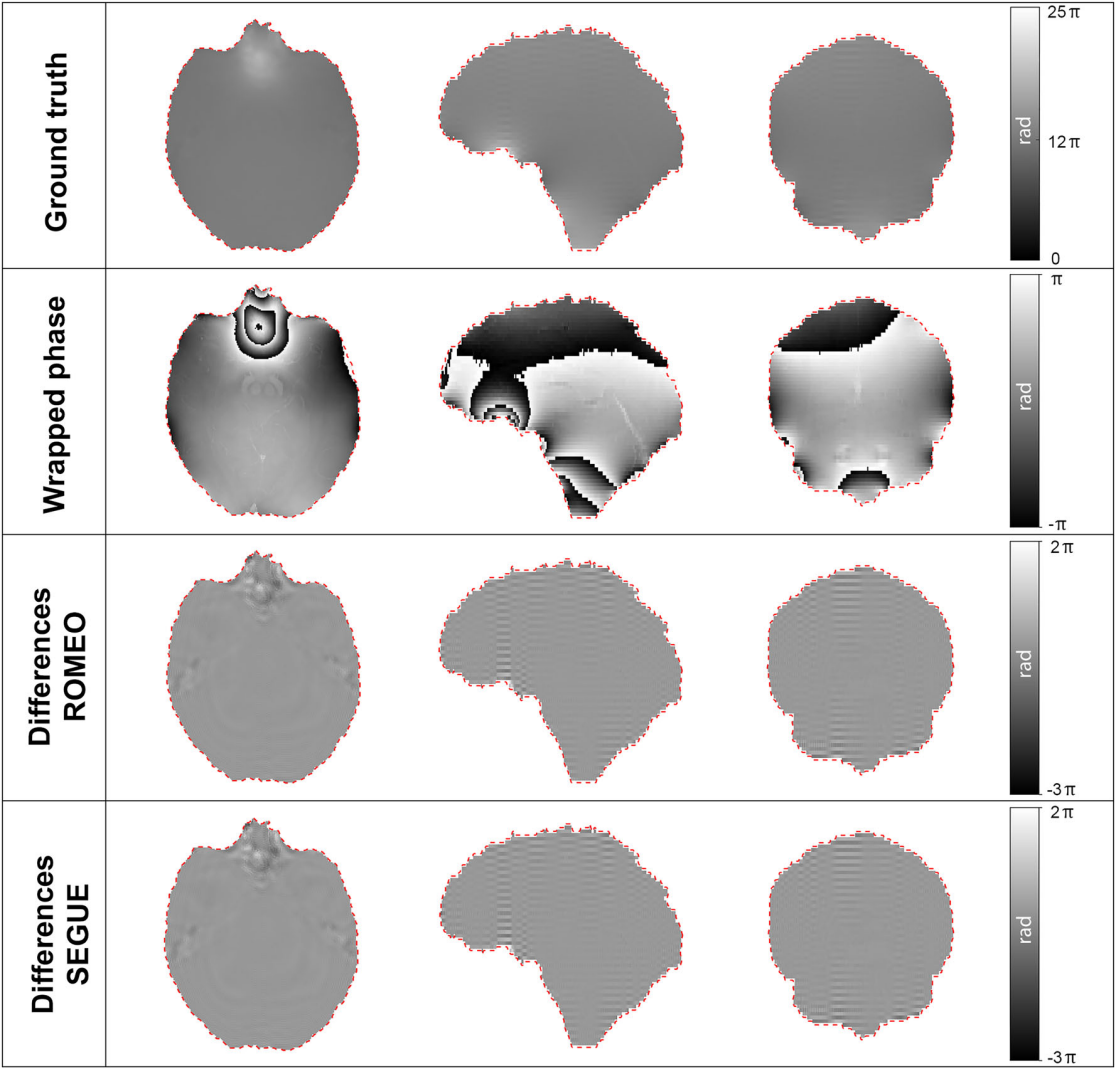
Phase unwrapping in the presence of a background field. The case corresponding to the second echo time (8 ms) is shown for illustrative purposes. The unwrapped phase is obtained from the simulated total field. The differences in radians between the estimated and expected phase maps for ROMEO and SEGUE are reported to highlight the spatial distribution of the errors.

Figure 3 and Table 1 show phase unwrapping results in the presence of a background field. Differences were on the order of tenths of a radian, with RMSE values of 0.9 and 1.1 rad in the skull‐adjacent region for ROMEO and SEGUE, respectively. Both methods were robust to noise (Supporting Information S7, Figure S3, and Table S2). ROMEO efficiency remained high even in the presence of complex geometries (Figure S4 and Table S3), where it demonstrated a ˜58‐fold speed advantage over SEGUE.

**TABLE 1:** Evaluation of phase unwrapping performance in the presence of background.

Method	MAE (rad)	MAE near skull (rad)	RMSE (rad)	RMSE skull (rad)	Time (s)
ROMEO	0.05	0.04	0.92	1.10	20
SEGUE	0.05	0.04	0.95	1.13	45

*Note*: RMSE, MAE, and computation time are reported for the whole brain and the skull‐adjacent brain ROI.

ROMEO's inclusion of an automated field map estimation step further enhances its versatility and ease of use, making it the preferred choice for the initial stage of QSM extraction.

### 4.2 Background Field Removal

**FIGURE 4:**
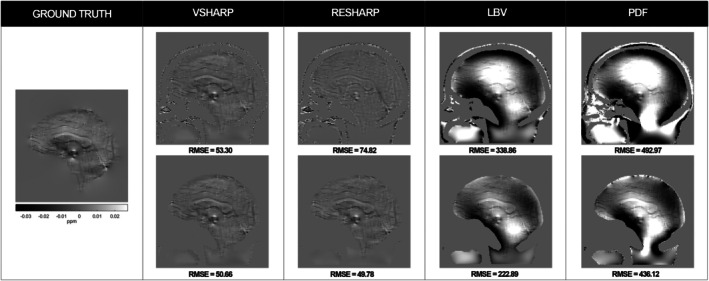
Sagittal representations of the local field estimated using different background field removal methods, compared to the ground truth. The first row shows results with minimal erosion; the second row corresponds to higher erosion (˜40%–45% voxel reduction within the ROI excluding bone and air), resulting from the automatic exclusion of voxels performed internally by each algorithm as a function of its specific parameters (e.g., kernel radius, peel depth). RMSE (in Hz) is calculated over a combined brain and GTV region. Detailed erosion percentages are reported in Figure 5.

The best performance over the entire ROI (i.e., brain and GTV) was observed by applying a fourth‐order polynomial after the RESHARP, LBV and PDF techniques, while a second‐order polynomial provided the best r esults for removing the residual field components after the VSHARP processing (Supporting Information S8 and Table S4). These results refer to the minimally eroded case. Figure 4 reports examples from both this condition and from stronger erosion (40%–50% voxel removal in brain and GTV), with VSHARP and RESHARP reaching convergence, conversely to LBV and PDF. Accordingly, RESHARP and VSHARP exhibited the best RMSE scores, with VSHARP obtaining the lowest RMSE of 53.31 Hz at minimal erosion (i.e., minimum kernel radius of one voxel).

**FIGURE 5:**
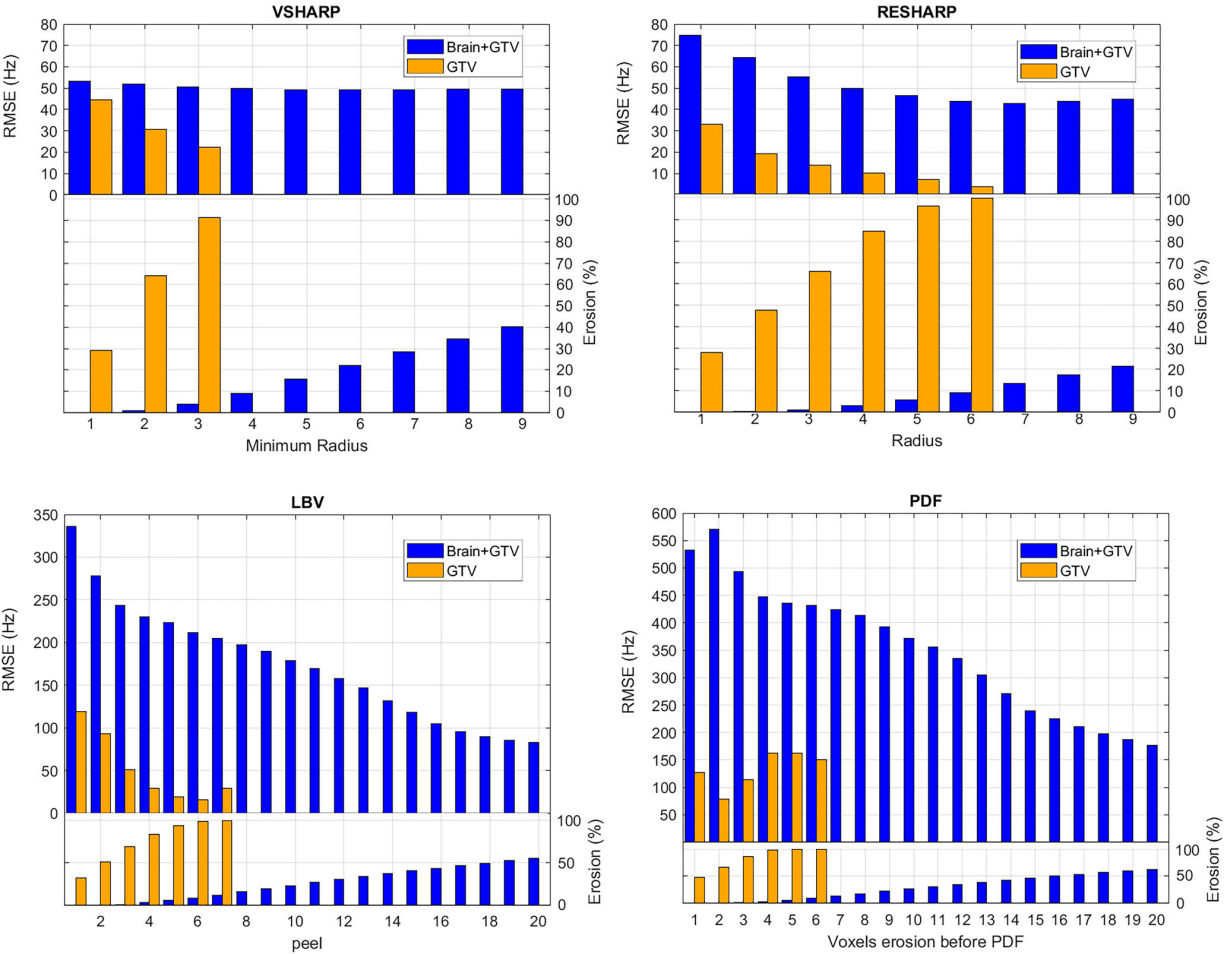
RMSE and the percentage of voxels eroded for the four background field removal techniques considered, as the parameters discussed in Section 3.1.3 are modified. Each parameter adjustment results in a corresponding change in RMSE and voxel erosion percentage.

RMSE values decreased as erosion increased, more markedly for LBV and PDF, while RESHARP and VSHARP showed minor variation (Figure 5). The orange bars highlight that, since the GTV was simulated near the boundary, even minimal erosion led to significant loss of GTV voxels, which increased with further erosion.

Given that RESHARP and VSHARP demonstrated the best performance, we further evaluated the impact of applying a small dilation before processing with these two algorithms to prevent direct erosion of the GTV. For VSHARP, expanding the mask by one voxel before applying minimal erosion resulted in an RMSE of 49.86 Hz within the GTV and maintained 53.31 Hz across the entire set of analyzed voxels. For RESHARP, the same expansion led to an RMSE of 54.93 Hz within the GTV and 74.90 Hz across the entire analyzed volume. Consequently, VSHARP was selected for the second step in the extraction of susceptibility maps.

The differences between the predicted and local ground truth after VSHARP and second order polynomial processing and the NRMSE for each anatomical component are reported in Supporting Information and Figure S5.

### 4.3 Dipole Field Inversion

**FIGURE 6:**
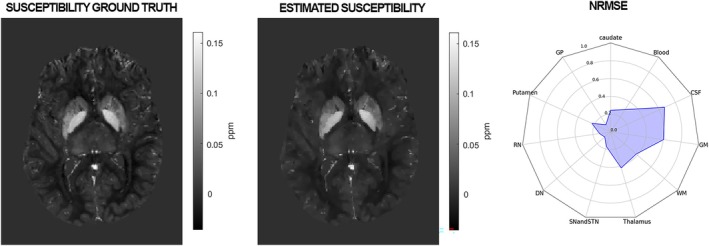
Expected versus predicted susceptibilities. Radar plot showing the normalized root mean square error (NRMSE).

After applying ROMEO unwrapping and the LN‐QSM on the soft tissues, we recorded a total RMSE on the cerebral available ROI of 38.36 ppm. The results are displayed in Figure 6, including the NRMSE for different cerebral components, while the corresponding MAE values are reported in Supporting Information S8 and Table S5. All individual brain structures exhibited a MAE below 4.1 × 10^−4^ ppm and a RMSE below 0.7 ppm; within deep gray matter regions specifically, the MAE remained below 0.05 × 10^−4^ ppm and the RMSE below 0.25 ppm.

### 4.4 In Vivo SBC Assessment

**FIGURE 7:**
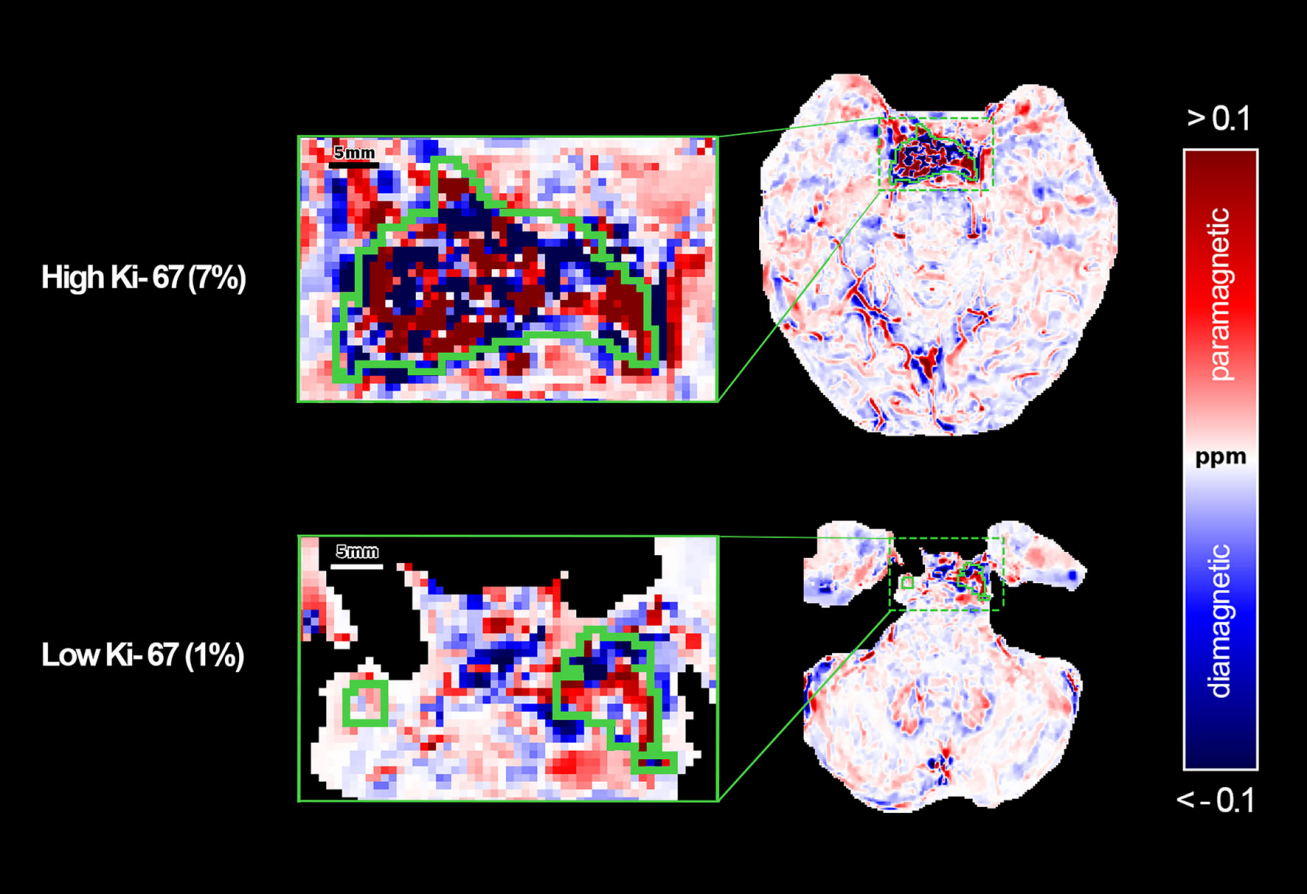
Comparison between a patient with low Ki‐67 and a patient with high Ki‐67. Color‐coded susceptibility map with values above and below threshold shown in red and blue, respectively. Tumors outlined in green. An inset detail aids visualization.

The workflow applied to in vivo data involved ROMEO algorithm for phase unwrapping, the VSHARP method for removing the background field, followed by a second order polynomial correction and LN‐QSM technique applied to local field to obtain the susceptibility in parts per million. Figure 7 presents a color map that allows easy distinction between paramagnetic and diamagnetic regions, with GTV contours overlaid for two patients with high and low Ki‐67 expression. Multiplanar views are also shown for a third patient (Supporting Information S9 and Figure S6).

Statistical measures of susceptibility variation highlighted the heterogeneity within the GTV. Specifically, the statistical test (Kruskal–Wallis test, alpha = 5%) revealed significant differences between GTV and WM, GM, CSF (Supporting Information S9 and Figure S7). Variables such as standard deviation, standard error of the mean, interquartile range, mean absolute variation, median absolute variation, kurtosis, percentile 25th and 75th showed *p*‐values lower than 0.05.

**FIGURE 8:**
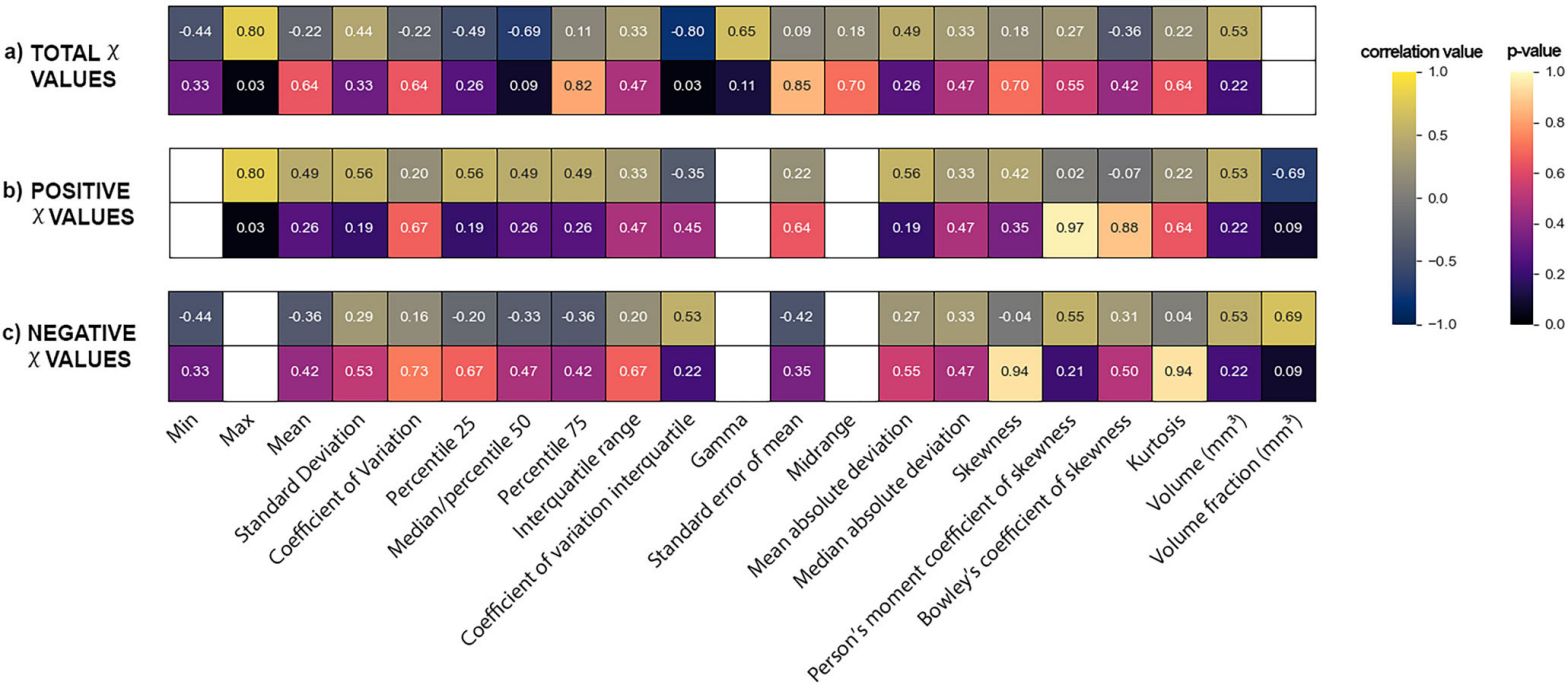
Heatmap of Spearman correlation for total, positive, and negative QSM values within the GTV. Correlation values and corresponding *p*‐values are represented with distinct color schemes.

A statistically significant correlation (*p* < 0.05) was observed between the maximum value and the Interquartile Coefficient of Variation (ICV) of the QSM values within the GTV with respect to Ki‐67, with a Spearman's coefficient of 0.8 and −0.8, respectively. The results of the statistical correlation analysis and the list of correlation coefficients and corresponding *p*‐values are presented in Figure 8.

In the classification model based on only two first‐order variables, Gradient Boosting and Random Forest achieved the best performance, with an accuracy of 85.7% (Table 2A). In contrast, Logistic Regression and SVM showed lower performance, with accuracy around 57%. In the extended model (Table 2B), which included a third automatically selected feature, Random Forest maintained an accuracy of 85.7%. The most relevant variables were maximum and ICV, with an additional contribution from gamma in tree‐based classifiers for the extended model.

**TABLE 2:** Classification performance of four machine learning models using two (A) or three (B) input features.

A					
Model	Accuracy	Precision (high/low)	Recall (high/low)	*F*1‐score (high/low)	Feature importance
Gradient boosting	0.86	1.00/0.80	0.67/1.00	0.80/0.89	Max: 0.42 ICV: 0.58
Logistic reg.	0.57	0.50/0.60	0.33/0.75	0.40/0.67	Max: 0.70 ICV: 0.30
Random forest	0.86	1.00/0.80	0.67/1.00	0.80/0.89	Max: 0.52 ICV: 0.48
SVM	0.57	0.50/0.60	0.33/0.75	0.40/0.67	Max: 0.64 ICV: 0.38

B					
Model	Accuracy	Precision (high/low)	Recall (high/low)	*F*1‐score (high/low)	Feature importance
Gradient boosting	0.57	0.50/0.60	0.33/0.75	0.40/0.67	Max: 0.41 ICV: 0.39 Gamma: 0.19
Logistic reg.	0.57	0.50/0.60	0.33/0.75	0.40/0.67	Max: 0.55 ICV: 0.23 Volume: 0.22
Random forest	0.86	1.00/0.80	0.67/1.00	0.80/0.89	Max: 0.34 ICV: 0.40 Gamma: 0.26
SVM	0.57	0.50/0.60	0.33/0.75	0.40/0.67	Max: 0.51 ICV: 0.25 Volume: 0.23

*Note*: The two baseline features used in both settings were selected based on their significant correlation with the Ki‐67 index (*p* < 0.05), showing high association with the target and low redundancy between them. In the extended models (B), a third feature was added based on RFE. Performance metrics include accuracy, precision, recall, *F*1‐score for each class (high/low), and feature importances.

## 5 Discussion and Conclusions

Chordoma is a rare and radioresistant tumor, typically treated with a partial surgical resection, followed by particle therapy. Preliminary studies have demonstrated that both sacral and SBC may contain hypoxic regions, potentially associated with radioresistance [6, 44]. In this study, we developed a QSM‐based pipeline to non‐invasively extract magnetic susceptibility in anatomically challenging skull base regions.

Each simulation was based on a realistic synthetic phantom from QSM Challenge 2.0. This anatomically accurate and ground‐truth controlled model was chosen to enable reproducible evaluation of algorithms performance under ideal conditions. Although Total Field Inversion methods [16, 45] could be exploited to directly invert the total field, we preferred to focus our pipeline on a classical three‐step QSM framework to enable step‐wise optimization and evaluation.

We evaluated SEGUE and ROMEO for phase unwrapping using a synthetic simulation of the skull base, with simulated MR signal acquisition including background fields to mimic clinical conditions. While ROMEO had been validated on complex topographic phantoms, it had not been tested in such anatomically realistic settings. ROMEO and SEGUE showed comparable unwrapping accuracy (RMSE of 0.9 and 1.1 rad near the skull; Figure 3, Table 1). In addition to its robust performance under noise (Supporting Information S7), ROMEO offers greater versatility, collectively supporting its choice as the most suitable method for our pipeline.

To evaluate background field removal, we extended the phantom to the whole head, incorporating a manually defined GTV at a typical skull base location (Figure 2d) based on the SBC dataset. Using the Forward Model, we simulated both local and background fields, accounting for strong susceptibility sources such as air cavities and skull bones. We tested RESHARP, VSHARP, LBV, and PDF, applying 3D polynomials and spherical harmonics (1st–4th order) to correct residual fields.

Since each method employs a distinct erosion process and the performance is closely dependent on the number of voxels removed, it was not possible to perform an equal comparison of the results. Therefore, we analyzed the trend of RMSE relative to the specific erosion parameters for each method, detailed in Section 3.1.3, considering both the GTV combined with brain regions and the GTV alone (Figure 5). The background field removal method that we selected was the algorithm that balanced the most accurate local field estimation within the GTV and whole brain, while preserving all GTV voxels after erosion (i.e., by expanding the GTV before applying the background removal, as in the in vivo application). VSHARP combined with a second order polynomial correction presented an RMSE of 53.31 Hz on brain and GTV and 44.40 Hz on GTV for minimal voxel canceling (6%). When applying an a priori dilation, an RMSE of 49.86 Hz was measured within the GTV, maintaining 53.31 Hz overall. Our QSM pipeline showed that susceptibility interfaces had limited impact on results, but precise ROI definition during background field removal remains critical. Although PDF and LBV performed well elsewhere, they failed to converge in our experiments, even after parameter tuning, unless susceptibility gradients were artificially reduced, which compromised realism. Given VSHARP's consistent performance under realistic, challenging conditions, it was selected for further analysis.

For dipole inversion, we selected the LN‐QSM technique, suited for whole‐head applications and integrated background field removal. While LN‐QSM could not reliably invert the total field (RMSE > 100 ppm), it performed well on the local field and was therefore used in the final QSM step. Combined with ROMEO on realistic, heterogeneous data, it achieved an RMSE of 38.36 ppm—comparable to top results from the Challenge 2.0 challenge [36] when the susceptibility ground truth was unavailable to participants (e.g., fifth‐place: 37.68 ppm). Consistently low MAE and NRMSE values across brain regions further confirmed its accuracy in estimating susceptibility tissue compartments.

Following the identified pipeline, QSM processing was applied to seven patients with SBC tumors. The cerebral susceptibility in healthy regions showed slight variations, in contrast to the GTV regions that revealed clear heterogeneity in patients (Supporting Information, Figure S7), as supported in the literature [46]. In our analysis we adopted a conservative definition of paramagnetic and diamagnetic regions based on the sign of the susceptibility values; future analysis could consider susceptibility source separation method for more reliable quantitative maps [47].

We analyzed both first‐order susceptibility features and volumetric characteristics, correlating them with the proliferative index Ki‐67. This analysis revealed a statistically significant correlation for both the QSM maximum value and the coefficient of variation interquartile with the Ki‐67. Ki‐67 is a well‐established cell proliferation marker widely used to assess tumor aggressiveness and predict outcomes [24, 48, 49], correlating with diffusion MRI features in SBC patients [42]. The biological determinants of the observed highly correlated maximum susceptibility values with Ki‐67 could be associated with heme iron, non‐heme iron, and calcium. Calcium, being diamagnetic, cannot explain the increased values observed [50]. Non‐heme iron is unlikely to accumulate in cranial chordomas through neuronal physiology; if present, it would most likely result from intratumoral hemorrhage, where degradation of extravasated blood could release iron, though such events seem to be rare in SBC [51]. A more plausible source is heme iron, particularly in the context of tumor‐associated hypoxia. In solid tumors, rapid proliferation increases the distance between cells and vessels, leading to oxygen deficiency [52, 53]. Thus, the observed correlation between Ki‐67 and maximum susceptibility in SBC may reflect elevated deoxyhemoglobin levels and hypoxic regions within the GTV [6]. Moreover, while increases in magnetic susceptibility may originate from post‐surgical blood residues or vascular structures, this explanation appears less consistent with our findings. No blooming artifacts were seen near the resection cavity, ruling out blood residues, and the signal lacked the organized morphology typical of venous vessels. To validate the hypothesis that the susceptibility signal is hypoxia‐related, future studies should nevertheless consider more specific histological markers, such as HIF‐1α [54], pimonidazole [55] or EF5 [56] rather than Ki‐67, while also disentangling the respective contributions of non‐heme iron, calcium, and oxygenation in QSM modeling.

Alongside correlation analysis, we developed a binary classifier to predict tumor proliferation from first‐order QSM features. Given the small sample size (*n* = 7), we limited input variables to the most strongly correlated features and used nested cross‐validation to reduce overfitting. Tree‐based models achieved 85.7% accuracy, consistent with their known ability to capture feature interactions and non‐linear dependencies [57]. Specifically, in the two‐feature model, maximum susceptibility emerged as the most important predictor, with an importance score of 0.52, supporting its potential link to hypoxia‐driven hyperproliferation.

While our preliminary results are encouraging, the rarity of SBC necessitates extending this study to a larger patient cohort to confirm statistical reliability. Further biological validation is required, either through specific histological markers (e.g., HIF‐1α) or via in vivo validation using hypoxia‐specific PET imaging, such as ^18^FMISO PET. We nevertheless believe that this work represents a promising starting point towards the non‐invasive characterization of SBC with the goal of advancing personalized treatments though hypoxia‐guided particle therapy [58].

## Supporting information


**Data S1:** mrm70193‐sup‐0001‐Supinfo.pdf.

## Data Availability

The data presented in this study are available upon reasonable request.
