# Supplementary material for: Quantitative Susceptibility Mapping in Skull Base Chordoma: In Silico Analysis and In Vivo Application Towards Indirect Hypoxia Assessment
Source: Magn Reson Med. 2025 Nov 24;95(4):2092–105. doi: 10.1002/mrm.70193 (PMC12850577; doi:10.1002/mrm.70193)
Supplement: Supplementary file 1 — Data S1: mrm70193‐sup‐0001‐Supinfo.pdf. [file MRM-95-2092-s001.pdf]

SUPPORTING INFORMATIONS

Theory

S1 Phase Unwrapping

ROMEO[1] is a path-following method that compute automatically a quality mask based on three weight products: spatial phase coherence, temporal phase coherence and magnitude coherence. The algorithm starts from the highest quality voxel and operates driven by a priority queue, constructing a spanning tree of the highest cost values based on Prim-Jarník algorithm.

PRELUDE[2] was considered the gold standard for the unwrapping of complex topography, although computationally demanding, especially for large-scale regions. In this context, SEGUE[3] takes shape as an effective alternative, since it is based on the same principles of PRELUDE, modifying the strategy of unwrapping. Consequently, SEGUE provides results comparable to those obtained with PRELUDE, but at rate of 1.5 to 70 times faster. Both methods adopt a partitioning technique, followed by a process of unwrapping and merging. In the case of SEGUE, the partitioning is handled by dividing the range $[0, 2\pi)$ into six subintervals. During this process, noisy voxels are excluded, if the phase variation exceeds 2π, and complex 3D regions are identified and connected. Once partitioning is complete, unwrapping starts from the region with the larger border, expanding to join neighboring regions.

S2 Background field removal

The traditional SHARP[4] technique assumes that background field is harmonic within the ROI and, as a result, satisfies the mean value property. The algorithm employes a truncated singular value decomposition (SVD) in the Fourier domain, to isolate the local field and reduce the noise while applying a constant convolution kernel to filter the background field. Both RESHARP and VSHARP enhance the original SHARP technique, each introducing unique improvements to optimize background field removal. RESHARP introduces a Tikhonov regularization process during the deconvolution to improve the residual field elimination. In contrast, VSHARP adopts a variable convolutional kernel within a predefined range.

The LBV method relies on the same assumption as SHARP, namely that background field is a harmonic function that satisfies the Laplace's equation. However, unlike SHARP, LBV addresses the problem by solving the boundary values problems of Laplace's equation, assuming simple boundary conditions to separate background local field from the local field.

In contrast, the PDF method is a non-parametric technique that assumes the magnetic field generated by a dipole outside the ROI is approximatively orthogonal to the field produced by a dipole inside the ROI. By applying the projection theorem in Hilbert space, the background field is decomposed into a field originating from external dipole.

Methods

S3 Dataset Reconstruction Challenge 2.0

A realistic digital phantom of the human head was developed based on high-resolution MRI images acquired at 7 Tesla[5], which were used to create a detailed ground truth including tissue segmentation and realistic maps of susceptibility, relaxation, and microstructure. Starting from this detailed model, k-space signal simulation was performed to generate synthetic data for controlled evaluation of QSM reconstruction algorithms.

Challenge participants were provided with synthetic multi-echo MRI data, including both magnitude and phase images[6]. Additionally, magnetic field maps and masks of brain regions of interest under background-free conditions were made available (Figure S1).

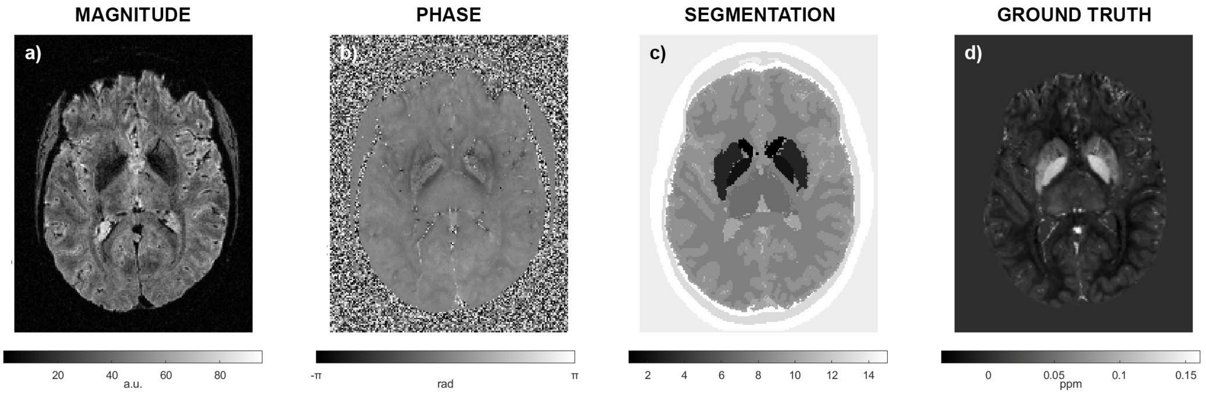

Figure S1 *Data from the challenge QSM 2.0. Axial views show (a) the magnitude from echo 4, (b) the phase from echo 4, (c) the segmentation of the head components, (d) the susceptibility ground truth provided.*

S4 Phase Unwrapping

In addition to simulations performed on the QSM challenge dataset, we assessed robustness and suitability of the phase unwrapping methods under two distinct conditions: noise resilience and complex topographies.

For noise robustness, we tested the algorithms on a synthetic in a 120×120 acquisition matrix of a circular region with a radius of 50 pixel, in which the phase changes linearly from -π to 6π. To simulate the impact of noise, we added Gaussian noise with varying standard deviations to both the real and imaginary channels, simulating different channel-specific signal-to-noise ratio (SNR) conditions (σ=1/SNR). The specific SNR levels included the conditions of absence of noise (SNR = ∞), low noise (SNR = 100), moderately high noise (SNR = 50), and elevated noise (SNR = 5).

To complement the phase unwrapping evaluation presented in the main manuscript, we employed an open-source dataset[7] characterized by complex topological features. This dataset,

previously employed in the original ROMEO publication, captures intricate spatial phase variations through irregular geometries and significant signal changes, including magnitude and ground truth phase images. The data consists of 256×256×256 volumes acquired at three different echo times. We wrapped the phase to simulate acquisition conditions. Using this challenging dataset, we compared the performance of ROMEO and SEGUE, with particular emphasis on differences in computational time and their ability to accurately process complex topographies.

S5 Supplementary Background Field Correction

To further reduce residual background field contributions, an additional correction step was applied after conventional background field removal, based on least-squares fitting using either 3D polynomials or spherical harmonics up to the fourth order. This choice was inspired by the approach from Shirai et al.[8], who proposed spherical harmonics fitting in combination with SMV filtering to suppress residual background fields without compromising the analysis mask. In their method, the residual field is modeled through a weighted least-squares problem:

$$argmin \left\| W(x,y,z)\left(B_{total}(x,y,z) - \sum_{l=0}^{L} \sum_{k=-K}^{K} A_{l,k} f_{l,k} \right) \right\|_2^2 \quad (1)$$

where $f_{l,k}$ are spherical harmonics expressed in Cartesian coordinates, $A_{l,k}$ are the coefficients to be estimated, and $W(x, y, z)$ is a spatial weighting function derived from the magnitude image.
We adapted this general framework, as implemented in the SEPIA[9] toolbox, and integrated it into our processing pipeline. In this adaptation, the least-squares fitting is restricted to voxels within a binary mask rather than using a continuous weighting function based on magnitude. SEPIA offers the flexibility to select the basis functions - either 3D polynomials or spherical harmonics up to fourth order - and this option was used based on empirical evidence gathered during application to our data.
This correction aims to remove structured residuals that may persist after standard background removal methods (e.g., V-SHARP, RESHARP, SHARP, LBV), potentially arising from sources such as B1 field inhomogeneities.

S6 Analysis on SBC patients

Features and related meaning are described in Table S1. During the analysis, we excluded the minimum QSM value for positive values ($\chi > 0$) and the maximum QSM value for negative values ($\chi < 0$), as these are expected to be zero in an ideal scenario for each patient. Additionally, the midrange and gamma (indicating the difference between the maximum and minimum) features were excluded due to their redundancy, as they provided overlapping information with the other features.

Concerning feature selection, as mentioned in the manuscript, we deliberately selected the two features showing the strongest correlation with Ki-67 (simple model). Importantly, these two

features were not correlated with each other, ensuring that they provided complementary, non-redundant information. This choice was made to guarantee a trade-off between interpretability, number of features, and available data. We then incorporated an additional feature (extended model) through automatic selection, with the purpose of exploring whether a controlled increase in model complexity could provide further predictive benefit while preserving robustness.

The extended model with a third feature encompasses a Recursive Feature Elimination (RFE) on the training folds (Figure S2). RFE is a feature selection technique that iteratively evaluates the predictive contribution of each variable and removes the least informative ones. In each iteration, a model is trained on the current set of features, and the importance of each feature is quantified according to the model. The least informative feature is eliminated, and the process repeats until the desired number of features is retained.

Concerning model development, we evaluated multiple algorithms to identify the most appropriate one, without assuming in advance which would yield the best performance, while excluding neural networks due to their need for larger datasets. Logistic regression aims to separate classes using a linear decision boundary. While Support Vector Machine (SVM) extend this approach by applying kernels to handle non-linear class separations. Random Forest and Gradient Boosting are instead tree-based methods that combine several decisions from multiple trees to capture complex relationship among features.

Model evaluation followed a nested cross-validation strategy, an approach to obtain unbiased estimates of model performance. In this framework, the dataset is first split in an outer loop (Fig.S2 left panel) using Leave-One-Out (LOO) cross-validation, in which each case is tested individually while the model is trained on all remaining samples. This procedure maximizes the use of limited data and provides nearly unbiased estimates of generalization. For each outer training set, an inner loop with 3-fold cross-validation is applied for hyperparameter tuning (Fig.S2 right panel) through Grid Search, which systematically explores a predefined set of parameter values and selects the configuration yielding the best performance within the training folds. Within the inner loop, the model is trained on a subset training set and evaluated on the remaining fold to select the best hyperparameters, ensuring that the outer test sample remains completely independent. Once the optimal hyperparameters are identified, the model is retrained on the entire outer training set and evaluated on the held-out outer test sample.

Feature	Meaning
Min	smallest value of the data
Max	largest value of the data
Mean	average of the data
Standard deviation	dispersion of the data from the mean
Coefficient of Variation	relative variability around the mean, in percentage
Percentile 25	value that gathers the first quarter of the data
Median or Percentile 50	central value that divides the sorted dataset into two equal halves
Percentile 75	value that gathers the third quarter of the data
Interquartile range	width of the central half of the data, calculated as the difference between the 75th percentile and the 25th percentile
Coefficient of variation interquartile	relative variability around the central value
Gamma	total range of values, given by the difference between the maximum and minimum
Standard error of the mean	quantification of the spread of sample means around the population mean
Midrange	central position of the data given by the average between minimum and maximum
Mean absolute deviation	measure of dispersion given by the mean of the absolute differences between each data point and the mean
Median absolute deviation	median of the absolute differences between each data point and the median
Skewness	measure of the asymmetry of the data distribution, indicating the degree and direction in which the distribution deviates from symmetry
Pearson's Moment Coefficient of Skewness	degree of asymmetry in a data distribution, computed by mean, median and standard deviation
Bowley's Coefficient of Skewness	measure of asymmetry in a data distribution, based on percentiles (75th, 25th) and the median
Kurtosis	measure of the shape of the distribution relative to a normal distribution
Volume (mm^3)	total volume calculated as the product of the spatial resolution and the number of voxels or regions analyzed
Volume Fraction (mm^3)	ratio of the negative or positive volume to the total volume

Table S1 *First-order and volumetric features included in the quantitative analysis, with their respective meaning.*

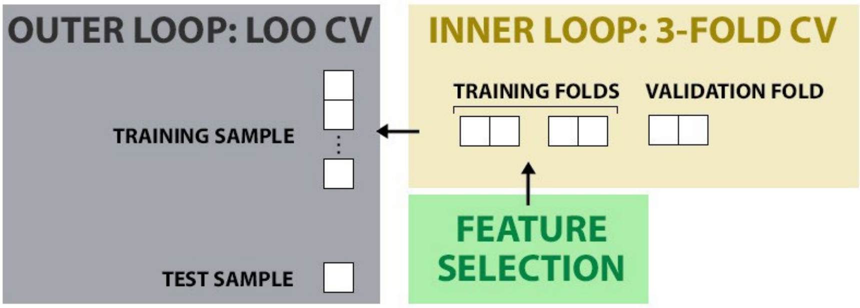

Figure S2 *Model workflow. The gray panel shows the outer loop with LOO cross-validation, holding out one sample as test at each iteration. The yellow panel shows the inner loop, where the outer training set is split into three folds for 3-fold cross-validation: two for training, one for validation. The green panel indicates feature selection is applied on the inner training folds when adding a third feature in the extended model.*

Results

S7 Phase Unwrapping

The two methods exhibited an effective unwrapping against different noise levels, reporting similar scores. Both methods performed properly and differences in RMSE were observed only at small order of magnitude, becoming evident in absence of noise (Tab.S2 and Fig.S3). RMSE grew with increasing noise, while the discrepancies between the predicted and reference phases became more evident.

**Method**	**SNR = ∞**	**SNR = 100**	**SNR = 50**	**SNR = 5**
SEGUE	$2.5 \cdot 10^{-6}$	$1.9 \cdot 10^{-1}$	$3.7 \cdot 10^{-1}$	3.9
ROMEO	$1.7 \cdot 10^{-15}$	$1.9 \cdot 10^{-1}$	$3.7 \cdot 10^{-1}$	3.9

Table S2 *Robustness analysis of the tested methods under increasing SNR levels. The table reports the full set of RMSE in radians obtained across different SNR conditions, while the corresponding figure S3 summarizes the results graphically to highlight performance trends. This evaluation was designed to assess the stability of each method in the presence of increasing noise.*

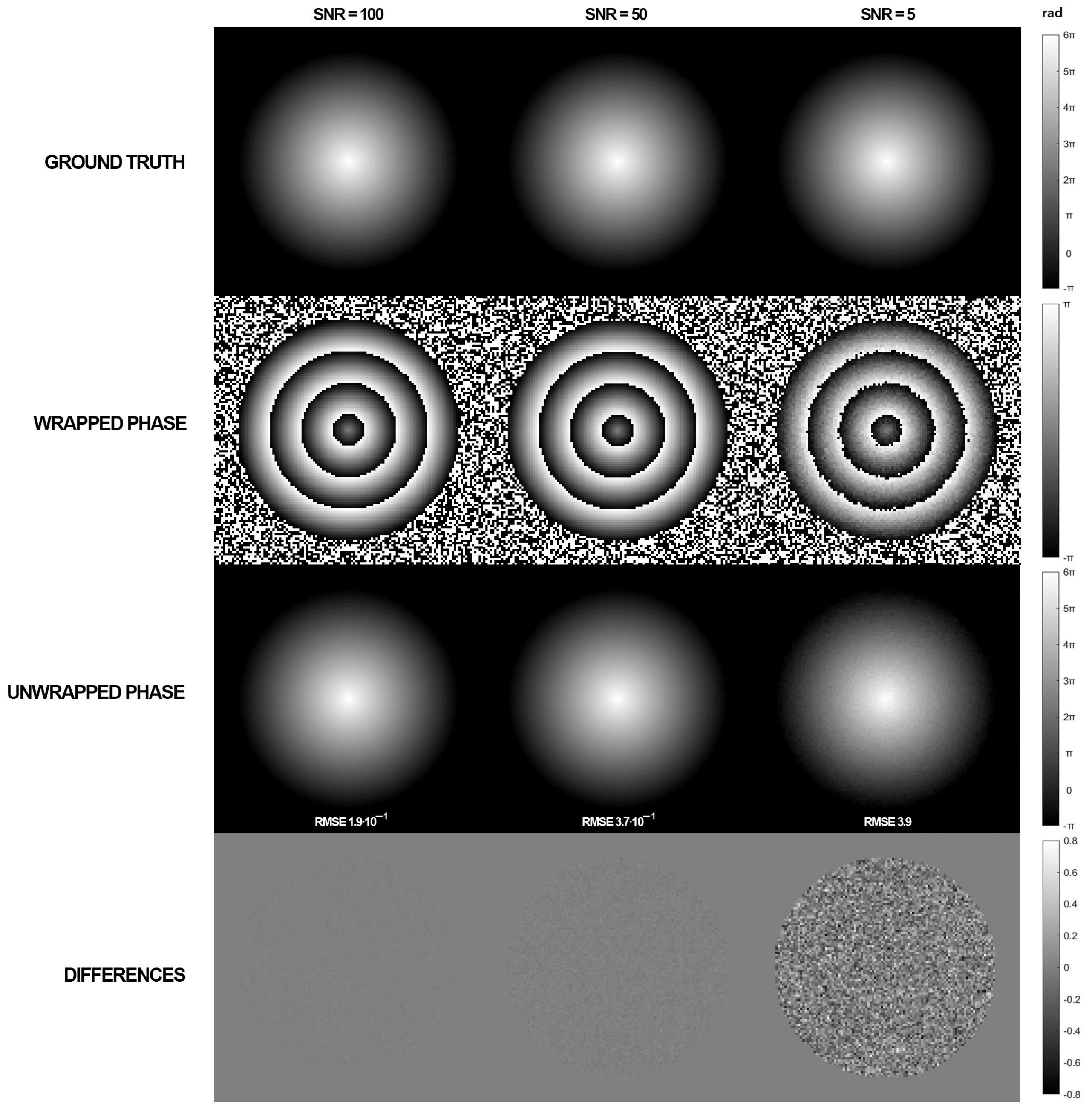

Figure S3 *Simulations of the circumference with increasing noise for ROMEO and SEGUE methods. The 1/SNR term represents the standard deviation of the noise added to the complex channel and RMSE is expressed radians. The figure shown refers to the results obtained with ROMEO; however, SEGUE yielded analogous results, as reported in Table S2.*

Both methods performed properly on topographic maps; however, ROMEO was considerably faster than SEGUE (about 58 times quicker, Table 2).

Method	Computation Time (hh:mm:ss)	Total RMSE (rad)
SEGUE	00:29:05	$2.9 \cdot 10^{-1}$
ROMEO	00:00:30	$1.2 \cdot 10^{-7}$

Table S3 *Performance of SEGUE and ROMEO on the 256 × 256 × 256 volume with three echo times, in absence of noise.*

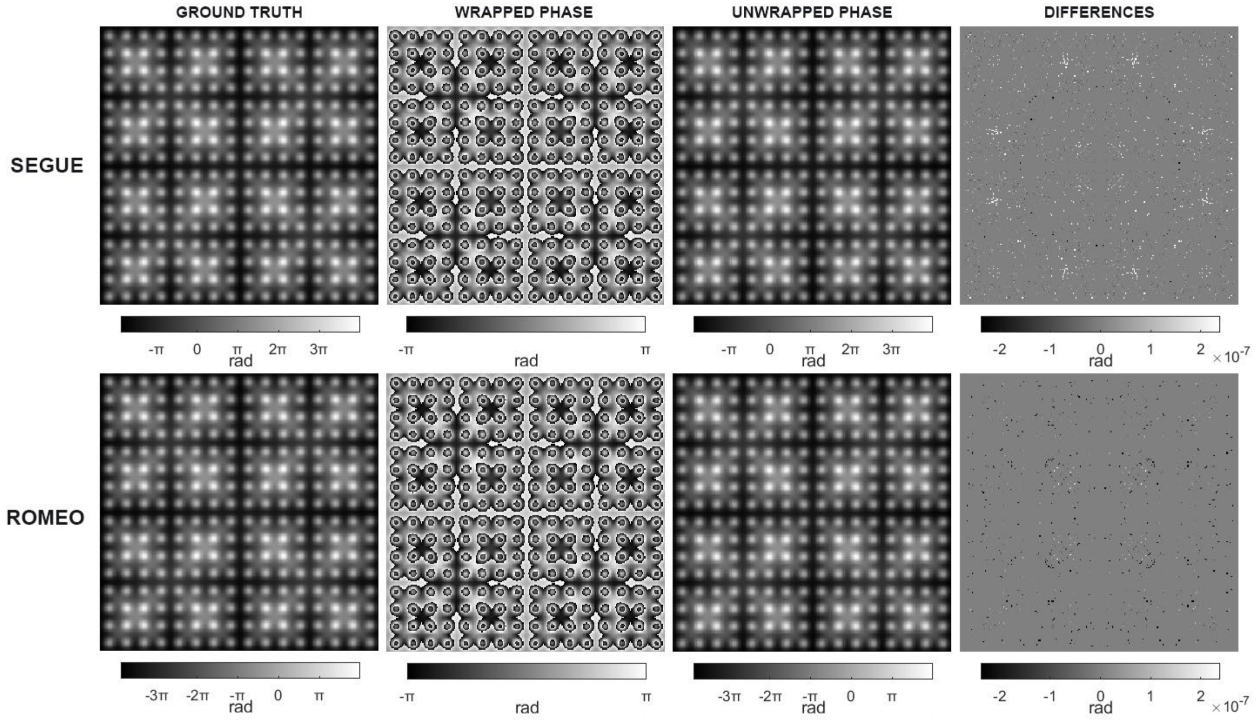

Figure S4 *Topographical simulations performing SEGUE and ROMEO unwrapping. The related RMSE values are reported in Table S3.*

S7 Background Field Removal and Dipole Field Inversion

	order	VSHARP		LBV		PDF		RESHARP	
		Brain and GTV	GTV	Brain and GTV	GTV	Brain and GTV	GTV	Brain and GTV	GTV
	0	58.06	46.73	1224.03	123.24	1183.56	94.64	75.68	33.68
**3D Polynomial**	1	57.85	47.28	1079.80	71.94	1183.29	94.34	75.91	33.80
	2	53.31	44.41	641.63	240.96	806.04	234.65	76.12	33.63
	3	53.92	46.57	421.30	109.14	745.02	149.17	75.70	33.40
	4	54.66	47.50	336.32	119.19	492.70	194.90	74.82	33.21
**Spherical Harmonic**	1	57.84	47.28	1079.76	71.94	1183.29	94.34	75.91	33.80
	2	56.24	47.55	1036.80	65.15	1183.50	94.46	76.16	34.01
	3	56.28	47.60	1028.44	57.74	1183.74	94.44	76.17	34.01
	4	56.40	47.61	1024.10	62.47	1183.56	95.38	75.94	33.90

Table S4 *Comparison of different field correction methods (VSHARP, LBV, PDF, and RESHARP) applied to both the whole brain and the GTV under the condition of minimum erosion. The table reports RMSE values in Hz for different polynomial and spherical harmonic correction orders.*

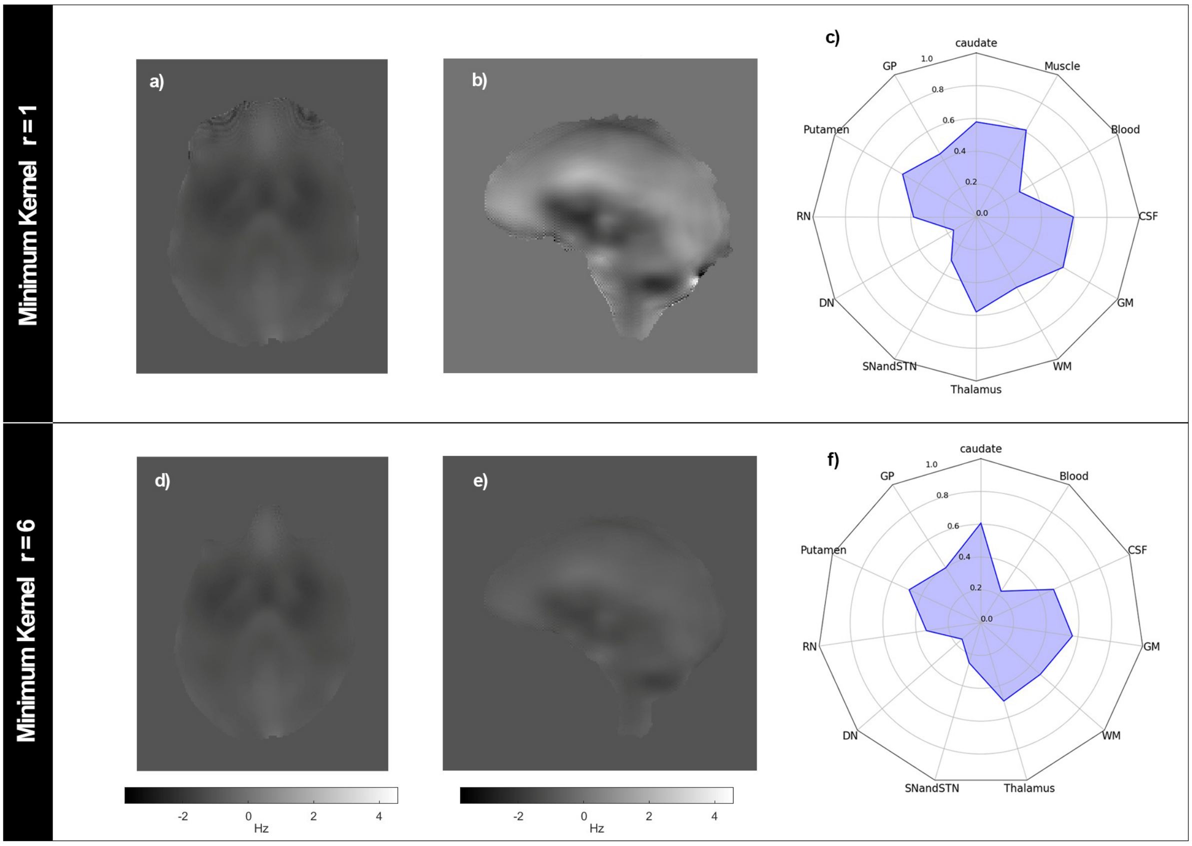

Figure S5 *Axial (a-d) and sagittal (b-e) views of the difference between the predicted and local ground truth after VSHARP processing with minimum kernel sizes of r=1 and r=6 (indicating different levels of erosion). The normalized root mean square error (NRMSE) for each component is shown in (c-f).*

**Component**	**MAE $\cdot 10^{-4}$ [ppm]**
Caudate	0.075
GP	0.043
Putamen	0.113
RN	0.006
SNandSTN	0.020
Thalamus	0.029
WM	0.115
GM	2.895
CSF	0.755
Blood	0.322

Table S5*. Mean absolute error (MAE) scores for the set of cerebral components, reported in parts per million (ppm).*

S8 Analysis on SBC patients

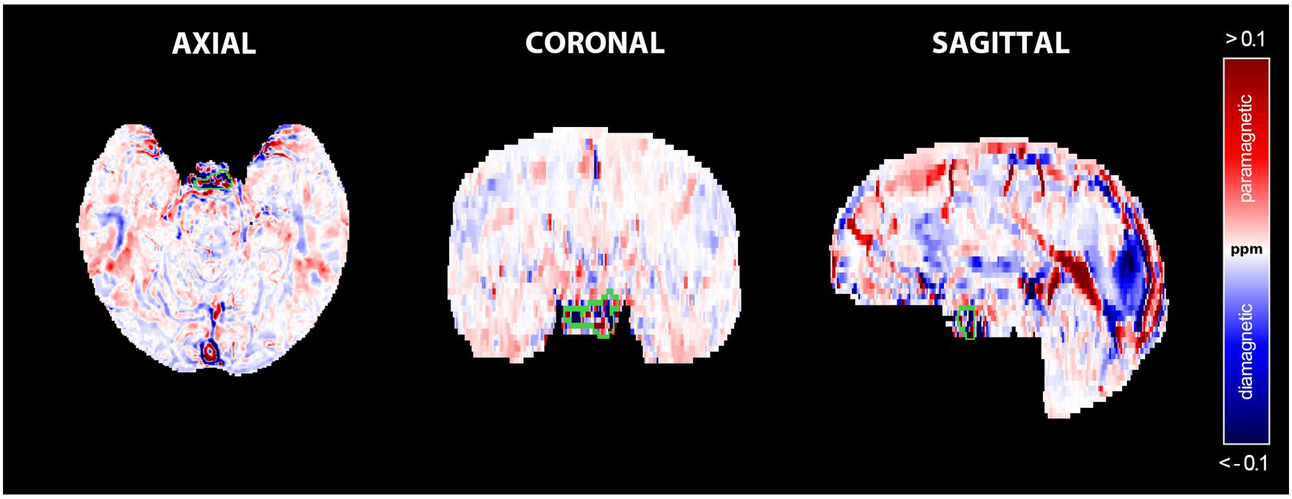

Figure S6 *Axial, sagittal and coronal views of a QSM color-encoded map of a SBC patient. The Gross Tumor Volume (GTV) is highlighted in green.*

a)

Feature	p-value
Mean	0.0253
Standard deviation	0.0006
Gamma	0.1320
Coefficient of variation	0.4712
Interquartile range (IQR)	0.0004
Coefficient of variation IQR	0.3602
Standard error of mean	0.0001
Mean absolute deviation	0.0004
Median absolute deviation	0.0004
Maximum	0.1793
Minimum	0.1382
Percentile 25	0.0002
Median	0.4022
Percentile 75	0.0012
Midrange	0.8545
Skewness	0.2120
Pearson's Moment Coefficient of Skewness	0.7315
Kurtosis	0.0008
Bowley's Coefficient of Skewness	0.0368

b)

Patient	Ki67 (%)
1	5
2	7
3	1
4	4
5	6
6	1
7	5

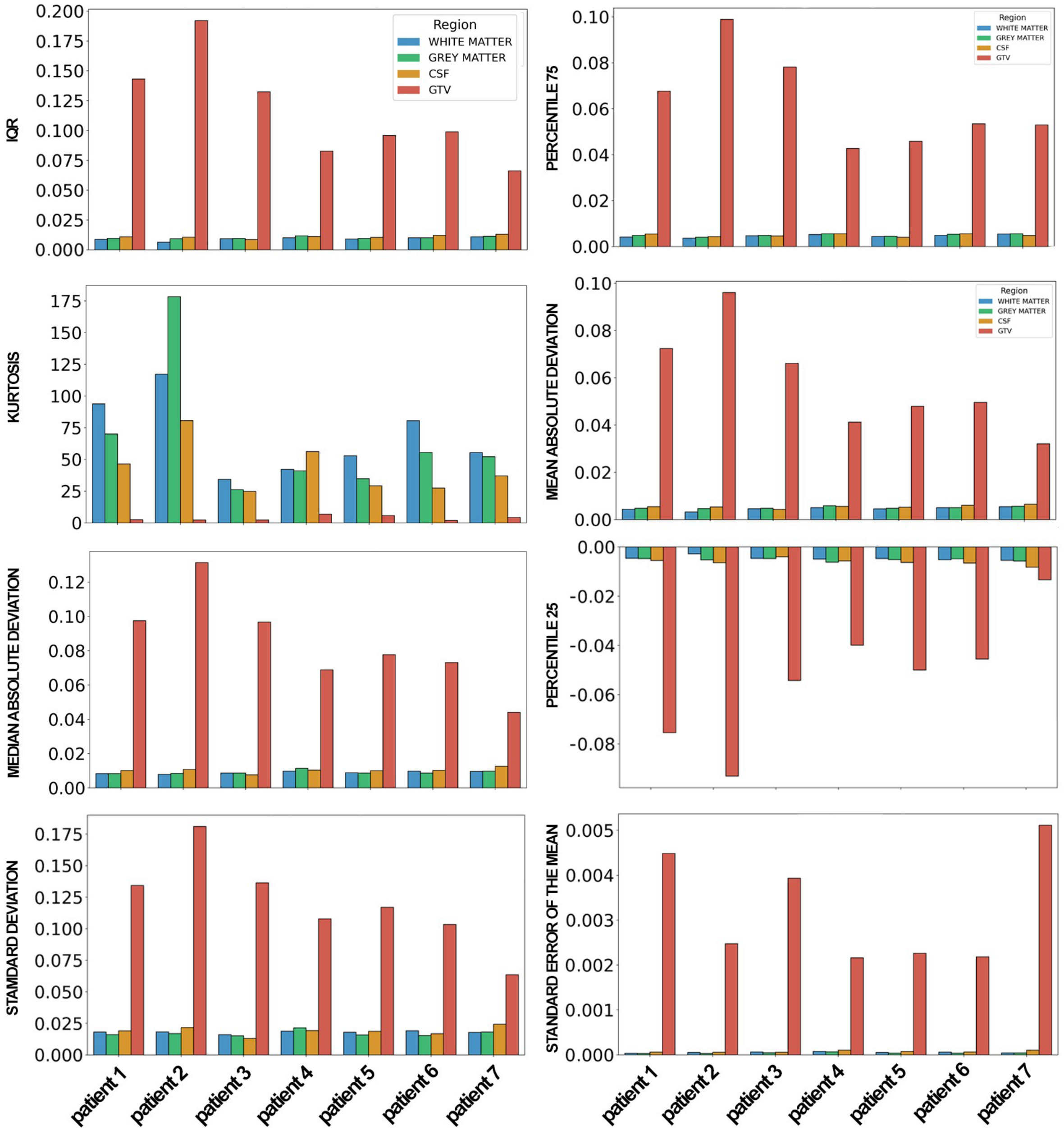

Figure S7 *a) Kruskal-Wallis test results comparing healthy Gray Matter, White Matter, Cerebro Spinal Fluid, and GTV across seven patients. The statistical significance of differences among regions is indicated by the p-values, highlighting features with significant variation. Histograms of the*

first-order features (ppm) are shown for features with p-value < 0.05, while panel (b) reports the corresponding Ki-67 values for patients.

References

1. Dymerska B, Eckstein K, Bachrata B, et al. Phase unwrapping with a rapid opensource minimum spanning tree algorithm (ROMEO). *Magn Reson Med*. 2021;85(4):2294-2308. doi:10.1002/mrm.28563

2. Jenkinson M. Fast, automated, N-dimensional phase-unwrapping algorithm. *Magn Reson Med*. 2003;49(1):193-197. doi:10.1002/mrm.10354

3. Karsa A, Shmueli K. SEGUE: A Speedy rEgion-Growing Algorithm for Unwrapping Estimated Phase. *IEEE Trans Med Imaging*. 2019;38(6):1347-1357. doi:10.1109/TMI.2018.2884093

4. Schweser F, Deistung A, Lehr BW, Reichenbach JR. Quantitative imaging of intrinsic magnetic tissue properties using MRI signal phase: An approach to in vivo brain iron metabolism? *Neuroimage*. 2011;54(4):2789-2807. doi:10.1016/j.neuroimage.2010.10.070

5. Marques JP, Meineke J, Milovic C, et al. QSM Reconstruction Challenge 2.0: a realistic in silico head phantom for MRI data simulation and evaluation of susceptibility mapping procedures. October 2020. doi:10.1101/2020.09.29.316836

6. Bilgic B, Langkammer C, Marques JP, Meineke J, Milovic C, Schweser F. QSM reconstruction challenge 2.0: Design and report of results. *Magn Reson Med*. 2021;86(3):1241-1255. doi:10.1002/mrm.28754

7. Dymerska B. *Simulation_complex_topography*. Harvard Dataverse, Version V3; 2020. doi:10.7910/DVN/HSSL3G

8. Shirai T, Sato R, Kawata Y, Bito Y, Ochi H. Region Expansion of Background Field Removal with Local Spherical Harmonics Approximation for Whole-brain Quantitative Susceptibility Mapping. *Magnetic Resonance in Medical Sciences*. 2023;22(4):497-514. doi:10.2463/mrms.mp.2021-0043

9. Chan KS, Marques JP. SEPIA—Susceptibility mapping pipeline tool for phase images. *Neuroimage*. 2021;227. doi:10.1016/j.neuroimage.2020.117611
